# Grow slowly, persist, dominate—Explaining beech dominance in a primeval forest

**DOI:** 10.1002/ece3.7800

**Published:** 2021-07-07

**Authors:** Roksolana Petrovska, Peter Brang, Arthur Gessler, Harald Bugmann, Martina Lena Hobi

**Affiliations:** ^1^ Swiss Federal Institute for Forest, Snow and Landscape Research WSL Birmensdorf Switzerland; ^2^ Forest Ecology Department of Environmental Systems Science ETH Zürich Zurich Switzerland

**Keywords:** Acer, AGR, dominance, Fagus, functional trait, LAR, NSC, regeneration, shade tolerance

## Abstract

Being able to persist in deep shade is an important characteristic of juvenile trees, often leading to a strong dominance of shade‐tolerant species in forests with low canopy turnover and a low disturbance rate. While leaf, growth, and storage traits are known to be key components of shade tolerance, their interplay during regeneration development and their influence on juveniles' survival time remains unclear. We assessed the ontogenetic effects of these three traits on the survival time of beech (*Fagus sylvatica*), and Norway and sycamore maples (*Acer pseudoplatanus*, *Acer platanoides*) in a primeval beech forest. Biomass allocation, age, and content of nonstructural carbohydrates (NSC) were measured in the stems and roots of 289 seedlings and saplings in high‐ and low‐vitality classes. Saplings experienced a trade‐off between absolute growth rate (AGR) and storage (NSC) as the leaf area ratio (LAR) decreases with biomass development. High LAR but low AGR and low NSC corresponded to beech with a marked ability to persist in deep shade while awaiting canopy release. In turn, a comparably small LAR in combination with a high AGR and higher storage (NSC), as observed in Norway maple and sycamore maple, reduced sapling survival time, thus offering an explanation for beech dominance and maple disappearance in the undergrowth of old‐growth beech forests.

## Graphical abstract

Can survival times of juvenile trees be inferred from traits underlying shade tolerance? This study analyzed the development of leaf, growth, and storage traits and their influence on the survival time of European beech, and Norway and sycamore maples in deep shade.

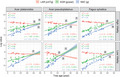

## 1 INTRODUCTION

In primeval monodominant *Fagus sylvatica* L. forests, seedlings (0–130 cm tall) and saplings (131–500 cm tall) often grow in deep shade for extended periods, due to low canopy turnover (Hobi et al., 2015; Runkle, 1985; Valverde & Silvertown, 1997). If canopy turnover is low (i.e., the mean time between recurring gap formation at any point in the forest), saplings that are able to persist for decades in shade are more likely to experience a canopy opening (i.e., a release event) enabling the subsequent promotion to canopy (Canham, 1985, 1990). Hence, high juvenile shade tolerance is pivotal in determining the survival time of *F. sylvatica* and may be compromised in co‐occurring species, explaining the low tree species diversity and *F. sylvatica* dominance frequently observed during succession (Korpel, 1995; Rey et al., 2019).

Shade tolerance can be assessed via functional traits, that is, morphological, physiological, and phenological features that reflect a species' ecological strategy (Pérez‐Harguindeguy et al., 2013). One of the concepts used to explain shade tolerance, the “carbon gain” hypothesis, postulates that saplings can enhance carbon gain in the shade, either by minimizing CO_2_ losses via respiration or by investing in the light‐harvesting capacity (greater leaf area and crown volume, Givnish, 1988) while maintaining a higher growth rate (Popma & Bongers, 1988; Walters & Reich, 1996). Another concept, the “defence and storage” hypothesis, relates shade tolerance to the resistance to herbivory, pathogens, and mechanical damage (Kitajima, 1994) and to storage (Kobe, 1997). Accordingly, shade‐tolerant species do not maximize growth in low light but invest a larger fraction of non−structural carbohydrates (NSC) in storage to buffer against stress during a prolonged period of shade (Kobe, 1997). The two concepts, “carbon gain” and “defence & storage,” are not mutually exclusive, but rather present different mechanisms of the complex phenomenon of shade tolerance.

The traits of shade tolerance change during ontogeny, that is, the development from seedlings to saplings. Maintenance and construction costs increase with tree height because the proportion of non−photosynthetic support tissue increases continually (Delagrange et al., 2004). At the same time, the ratio of leaf area to total tree biomass (LAR) diminishes as young deciduous trees grow (Niinemets, 1998), and thus, the leaf area capacity may be limited in terms of providing photosynthates for both growth and storage. NSC dynamics in juvenile regeneration during ontogenetic development are not well understood (Hartmann et al., 2018). Although the NSC concentration is expected to decrease with tree height (Machado & Reich, 2006), the growing volume of support tissue suggests that allocation to storage increases in proportion to plant mass (Plavcová et al., 2016), which in turn may decrease growth under carbon limitation (Wiley & Helliker, 2012). Thus, during ontogenetic development, young trees may experience a trade‐off between growth and storage, leading to a shorter survival time.

Studies on the relationships among the traits of shade tolerance in broad‐leaved species have mostly been focused on seedlings in garden experiments (Gibert et al., 2016) and have not involved investigations of how leaf, growth, and storage traits develop with age, thus leading to patchy evidence (Valladares et al., 2016; Valladares & Niinemets, 2008). Moreover, we are not aware of any existing study on deciduous trees addressing how ontogenetic changes in traits of shade tolerance may affect the survival time of juvenile trees. We define “regeneration survival time” as the potential time that seedlings and saplings can survive in the unfavorable environment of deep shade, which corresponds to the time until the first canopy release. In the present study, we aimed to combine leaf, growth, and storage traits as proxies of shade tolerance to infer the survival time of juvenile beech and co‐occurring species.

Due to low tree diversity in monodominant *F. sylvatica* forests, we studied its seedlings and saplings (0–5 m height) and the most abundant competitor species, such as *Acer pseudoplatanus* and *Acer platanoides*, in two vitality classes. Comparisons of traits between trees with high and low vitality (i.e., the capacity to grow, resist stress, and acclimate to environmental conditions; adapted from Brang, 1998; Dobbertin, 2005) made it possible to relate trait performance to survival time. In particular, we investigated the following research questions: (a) Which traits relating to leaf, growth, and storage can discriminate between low‐ and high‐vitality regeneration? (b) Is there a trade‐off between growth and storage traits among species of low and high vitality? (c) How do these traits affect regeneration survival time?

## 2 MATERIALS AND METHODS

### 2.1 Study area and plot selection

The Uholka‐Shyrokyi Luh reserve in Ukraine belongs to one of the most investigated *F. sylvatica*‐dominated primeval forests of Europe and is listed as a UNESCO World Heritage site (Stillhard et al., 2019; Trotsiuk et al., 2012; Zenner et al., 2020). In this study, we focused on the Uholka part of the forest (coordinates: 48°16′N, 23°40′E), which was selected because it has a greater share of *Acer* spp. than in the Shyrokyi Luh part of the reserve. The Uholka part covers 4,729 ha, ranging from 400 to 1,300 m a.s.l., with a mean annual temperature of about 8°C (−3°C in January and 18°C in July at 430 m latitude) and a mean annual precipitation of 1,134 mm (Commarmot et al., 2013). *F. sylvatica* is abundant in the regeneration, constituting 83%–97% in density for the height classes 10–129 cm and ≥130 cm up to 5.9 cm DBH (diameter at breast height, i.e., 1.3 m; Commarmot et al., 2013). The share of *A. pseudoplatanus* shrinks from 15% in the 10–39.9 cm height class to 3% in the 3–3.9 cm DBH class, while the share of *A. platanoides* is almost zero for trees with a DBH >2 cm (inventory 2010, analysis not shown). The forest is dominated by a small‐scale disturbance regime with a mosaic of mainly small canopy gaps (98% are <200 m^2^); only a few large, stand‐replacing events were detected in a study using high‐resolution satellite imagery (Hobi et al., 2015).

We randomly selected six plots (total area 2.53 ha) varying from 0.2 to 0.7 ha in size, in which mixed regeneration of the three species was present in subplots. Within the six plots, nine subplots from 140 to 520 m^2^ (total 0.26 ha) were delineated to contain as many seedlings/saplings of the target species/sizes/vitality classes as possible. Among this regeneration, 289 target seedlings and saplings were randomly selected and marked according to the following criteria: three species (*F. sylvatica*, *A. pseudoplatanus, and A. platanoides*), two vitality classes (low and high), and eight height classes: 0–10, 11–20, 21–35, 36–60, 61–90, and 91–130 cm as seedlings, 131–200 and 201–500 cm as saplings, one seedling/sapling per plot in each height and vitality class (see Table S1). Browsing was apparent on all plots, with many recovered *Acer* spp. trees having scars, while *F. sylvatica* regeneration was almost untouched.

### 2.2 Classification into vitality classes

We developed criteria for juvenile trees based on the vitality assessment used for adult trees, in which tree crowns are assessed visually (Eichhorn et al., 2016; Roloff, 1991) and growth is measured in the field (Dobbertin, 2005). Crown transparency has been shown to correlate well with relative growth rate (Lorenz et al., 2004; Solberg, 1999) and also with subsequent tree mortality and survival (Dobbertin & Brang, 2001; Schmid‐Haas, 1993). Hence, we classified seedlings and saplings (Table 1), taking into account crown transparency (leaf loss and/or dieback) and the increment of the apical shoot for several years, but we used the branching pattern and stem condition as additional discriminators to differentiate between high‐ and low‐vitality trees (Collet et al., 2011; Roloff et al., 2016). To avoid an inconsistent crown transparency assessment (Dobbertin, 2005), only one evaluator assessed all seedlings and saplings, using site‐specific reference trees. A reference tree is a tree with full foliage (defoliation 0%) that grows at a particular site, considering altitude/latitude, site conditions, and social status (Eichhorn et al., 2016). Trees browsed during the current season were not considered.

**TABLE 1:** Criteria used to classify juvenile trees into high‐ and low‐vitality classes based on (a) crown transparency, (b) apical shoot increment, (c) branching pattern, and (d) stem damage

Parameter	High vitality	Low vitality
Crown transparency (leaf loss, crown dieback)	Leaf loss <20% No dead branches and no crown dieback	Leaf loss >20% Dead branches or crown dieback
Apical shoot increment for several years	Large apical increments for 3–5 years Browsed in the past but recovered	Small apical increments for 3–5 years Browsed in the past and not recovered
Branching pattern	Vigorous branching	Degenerative branching
Stem damage	Intact stem, no diseases	Scars, bacterial/fungi diseases

### 2.3 Measurements and calculations

#### 2.3.1 Field measurements

The following measurements were taken before tree excavation: diameter at root collar (DRC), tree height, and height of the crown base (height of the lowest foliage, excluding epicormic shoots). We measured crown area projection by two perpendicular crown diameters using a pendulum suspended from the outermost branches to the ground. Stem height increment was measured for the most recent 5–10 years (until the last visible bud scale scar) to the nearest millimeter. Leaf area index and indirect site factor (ISF), that is, the proportion of diffuse solar radiation at a given location relative to that in the open, were assessed with hemispherical photographs (Coolpix 4500, Nikon, Japan) with a 183° fish‐eye lens (Nikon FC‐E8) mounted on a tripod (Thimonier et al., 2010). Photographs were taken just above the uppermost leaves of every tree, bending saplings taller than 1.5 m to allow photograph shooting of canopy. We then excavated trees manually and cleaned roots with water to avoid damage to the fine roots.

#### 2.3.2 Postharvest processing (mid‐May to mid‐July)

The sampled trees were separated at the root collar into aboveground biomass (foliage, stem, branches) and belowground biomass (roots). Pieces of 5‐cm length from the stem at the level of the root collar and from the coarse roots (diameter >2 mm) were cut for NSC analysis and placed in a microwave at 900 W twice for 15 s immediately after the harvest (Popp et al., 1996). In the case of seedlings without coarse roots, we used the taproot. All fresh leaves per tree were scanned with a smartphone (Petiole, version 2.0.1, Petiole Ltd. 2019) after calibration of the camera. Foliage, stems, branches, and roots were dried at 65°C for 3 days until a constant weight was reached and then weighed to the nearest 0.01 g.

#### 2.3.3 Calculations

Crown area projection was calculated based on the quadratic mean radius (Pretzsch et al., 2015). We multiplied crown area projection by the difference between tree height and the height of the crown base to obtain crown volume (assuming it is a cylinder). Hemispherical photographs were analyzed with the program Hemisfer (version 2.2, ©Patrick Schleppi, WSL). ISF was estimated using the method introduced by Thimonier et al. (2010). We calculated the trait variables according to the formulae in Table 2 and present the final results in Table S1.

**TABLE 2:** Variables calculated for leaf and growth traits

Traits	Variable	Formula	Units
Leaf	Leaf area ratio, LAR	$\text{LAR}=\frac{\text{LA}}{m_{\text{total}}}$(1) where LA is the leaf area per tree and $m_{\text{total}}$ is the total dry mass per tree	cm^2^/g
	Specific leaf area, SLA	$\text{SLA}=\frac{\text{LA}}{m_{\text{leaf}}}$ (2) where LA is the leaf area per tree and $m_{\text{leaf}}$ is the total leaf dry mass per tree	cm^2^/g
	Leaf mass fraction, LMF	$\text{LMF}=\frac{m_{\text{leaf}}}{m_{\text{total}}}$ (3) where $m_{\text{leaf}}$ is the total leaf dry mass per tree and $m_{\text{total}}$ is the total dry mass per tree	
	Crown area	$p=\bar{r^{2}}\pi$ (4) where $\bar{r}=\sqrt{\frac{\left(r_{1}^{2}+r_{2}^{2}+r_{3}^{2}+r_{4}^{2}\right)}{4}}$ and *r*_1_…*r*_4_ are radii in four cardinal directions	m^2^
Growth	Absolute growth rate, AGR	$\text{AGR}=\frac{m_{\text{total}}}{t}$ (5) where $m_{\text{total}}$ is the total dry mass per tree and *t* is tree age	g/year
	Shoot mass growth rate (leaves + branches)	$r_{\text{shoot}}=\frac{m_{\text{shoot}}}{t}$ (6) where $m_{\text{shoot}}$ is the total dry shoot mass per tree and *t* is tree age	g/year

### 2.4 NSC analysis

Non−structural carbohydrates (NSC) represent the storage trait in the “defence and storage” concept of our study; they are sugars of low molecular weight (glucose, fructose, and sucrose) and starch. NSCs were analyzed according to the Wong (1990) protocol modified by Hoch et al. (2003). The harvested (mid‐May to mid‐July) coarse root sections of larger saplings (2–4 cm DRC) were limited to 5–10 mm diameter and the harvested stem sections to 10 mm of wood directly under the bark. The milled stem sections (without bark) and the root (without bark if possible) of each sapling (10–12 mg) were boiled in 2 ml of distilled water for 30 min. After centrifugation, we added invertase and isomerase (baker's yeast; Sigma‐Aldrich) to an aliquot of 200 μl to degrade sucrose and convert fructose into glucose. After enzymatic conversion to gluconate‐6‐phosphate with the hexokinase reaction (hexokinase produced by Sigma Diagnostics), the total amount of glucose (sugars) was determined photometrically at 340 nm in a 96‐well microplate photometer (HR 7000; Hamilton). We took 500 μl of the extract (including sugars and starch) and incubated it with a fungal amyloglucosidase from *Aspergillus niger* (Sigma‐Aldrich) for 15 hr at 49°C to break starch into glucose. Total glucose (corresponding to NSC) was determined photometrically as described above. The concentration of starch was calculated as NSC concentration minus the free sugar concentration determined in the first step. Standards of pure starch and glucose, fructose, and sucrose solutions were used as controls, and standard plant powder (orchard leaves; Leco) was included to test the reproducibility of the extraction. NSC concentrations were expressed on a gram per dry matter basis and scaled to the whole stem and root dry biomass to obtain the absolute value of total NSCs pool per tree. We assumed no large vertical or horizontal NSC gradient within the wood (which is all sapwood); therefore, upscaling to the whole stem and dry root mass should give realistic absolute values for the NSC content per tree. We ran the analysis in the same laboratory with no change in protocol (Quentin et al., 2015).

### 2.5 Dendrochronological analysis

From each harvested tree, a stem disk was cut at the level of the root collar using a microtome to determine age and radial growth. The stained disks were photographed (Canon EOS 700D) and analyzed with WinDENDRO™ (Regent Instruments Inc.) under a microscope. The number and width of the rings were measured in 2–4 perpendicular directions because of the eccentric tree piths and then arithmetically averaged.

### 2.6 Statistical analysis

#### 2.6.1 Variable selection and discrimination between vitality classes

Principal component analysis (PCA) was used to select variables among leaf and growth traits with the highest contribution to principal components (Figure S1, Tables S2). The comparison of means among vitality classes (Table S3) was made with Yuen's trimmed *t* test (Yuen, 1974) with the Benjamini–Hochberg *p*‐value adjustment, with the significance level set to 0.05 (Benjamini & Hochberg, 1995); and among species with a heteroscedastic two‐way factorial ANOVA (Figure S2) based on trimmed means (20% trimming level). This procedure downplays outliers, heavy‐tailed distributions, and unequal sample sizes and is robust against violations of homogeneity. We waived the violation of normality because our sample size exceeded 50 observations.

#### 2.6.2 Effect of biomass partitioning on traits of shade tolerance

We modeled the influence of biomass partitioning to leaves and shoots on traits of shade tolerance such as LAR, AGR, and NSC with multivariate analysis of covariance MANCOVA (R package *car*), the Pillai–Bartlett trace test, and ANOVA type III for unbalanced designs (details in S1.1). The multivariate linear regression is able to capture linear growth observed in young trees within selected height classes and to model the simultaneous influence of explanatory variables such as species, tree age, LMF, and shoot growth rate on the response variables LAR, AGR, and NSC (Equation 1). The pairwise comparison of species' means (R package *emmeans)* was made with a post hoc Tukey honest significant difference or Tukey HSD test (Tukey, 1949).

(1) $$\left\{\begin{array}{c} y_{1}=\ln\left(\text{LAR}\right)\\ y_{2}=\ln\left(\text{AGR}\right)\\ y_{3}=\ln\left(\text{NSC}\right) \end{array}\right.=\beta_{0}+\beta_{1}\text{species}+\beta_{2}\;\ln\left(\text{LMF}\right)+\beta_{3}\;\ln\left(\text{shoot growth}\right)*\text{age}+\beta_{4}\;\ln\left(\text{shoot growth}\right)+\beta_{5}\;\text{age}+\varepsilon$$

where ε is the error term that follows a standard normal distribution (Figure S3). All continuous variables were log‐transformed, except for tree age, and centered. The assumption of homogeneity of covariance (Box's *M* test at *α* < 0.001)) was not violated (*p* = 0.03). Correlation between the covariates was moderate: *r* = 0.50 between shoot growth rate and tree age, low correlation *r* = −0.11 between shoot growth rate and LMF, and *r* = −0.36 between LMF and age. Multicollinearity was tested with variance inflation factor VIF (R package *olsrr*) and did not exceed 3, indicating low‐to‐moderate multicollinearity. The analyses and visualization were run in R, version 3.6.1 (R Core Team, 2019).

## 3 RESULTS

### 3.1 Biomass allocation

**FIGURE 1:**
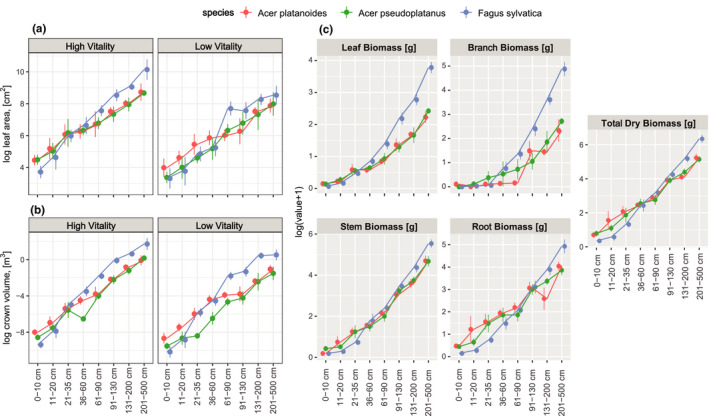
Panel a: mean leaf area; panel b: mean crown volume according to species and height class for trees of high and low vitality. Panel c: mean leaf, branch, stem, root, and total dry biomass of the high‐vitality trees in height classes: Dots represent means and whiskers represent standard errors

In deep shade (mean ISF 1.95%–3.34% on the six plots, Table S1), *F. sylvatica* invested more heavily in leaf area development and crown volume starting from the height class >36 cm in both high‐ and low‐vitality trees than its competitor species, while its mean leaf area and crown volume were smaller than those of the competitors for smaller seedlings <36 cm height (Figure 1). Mean biomass allocation to leaves increased with tree height for all species, with *F. sylvatica* investing more in leaves than *Acer* spp. above a tree height of 60 cm (Figure 1). Patterns in branch biomass allocation were similar to those for leaf biomass, but maple seedlings <60 cm tall generally did not grow branches if not browsed. Allocation to the stem was more similar in the three species than allocation to leaves and branches. Mean biomass partitioning to roots was higher in *Acer* spp. up to a height of 90 cm. In short, *Acer* spp. focused on conservative investments in the stem and roots, while *F. sylvatica* pursued light‐harvesting and space occupation strategies by allocating biomass to leaves and branches.

As expected, species identity significantly affected leaf traits such as leaf area (*p* = 0.018) and crown volume (*p* = 0.025) in the heteroscedastic two‐way factorial ANOVA where the two vitality classes and two height classes were pooled together (Figure S2). The species effect was also significant for the growth trait shoot biomass (leaf and branch mass, *p* = 0.004), but not for total dry mass (*p* = 0.335). The total mean biomass of *Acer* spp. trees (39 ± 6.7 g for *A. pseudoplatanus* and 44 ± 8.4 g for *A. platanoides*) was smaller, although not significantly so, than that of *F. sylvatica* (86 ± 17 g, mean ± *SE*). The three species allocated biomass differently: *F. sylvatica* had a significantly larger leaf area (3,320 ± 39 cm^2^) than *A. pseudoplatanus* (1,170 ± 179 cm^2^, *p* = 0.046) and *A. platanoides* (1,516 ± 267 cm^2^, *p* = 0.07), a larger crown volume (1.0 ± 0.3 m^3^, *p* = 0.029) than both *Acer* spp. (0.1 ± 0.0 m^3^ for *A. pseudoplatanus* and 0.2 ± 0.1 m^3^ for *A. platanoides*), and a much greater shoot (leaf + branch) biomass (21 ± 5 g, *p* = 0.006) than the two competitors (4.6 ± 0.7 g for *A. platanoides* and 4.9 ± 0.9 g for *A. pseudoplatanus*).

### 3.2 Discrimination between vitality classes

Leaf traits (LAR, leaf area, crown projection area, crown volume), together with growth traits (AGR, root collar diameter, mean height increment; growth rates of leaf, stem, roots, and shoots biomass per year) and storage (NSC), contributed 56.6% to the first axis in the PCA, while LAR, LMF, SLA, and tree ring width contributed 13.6% to the second axis, summing to 70.1% of the total variance in the tree traits. The major contributors to the two principal components were AGR (11.0%), NSC pool (7.6%), LAR (32.6%), and LMF (33.0%) (Figure S1, Table S2). High‐vitality trees differed from low‐vitality individuals in that they had a significantly higher LAR (t(205.71) = 4.92, *p* < 0.001), LMF (t(219.37) = 5.88, *p* < 0.001), and NSC (t(178.60) = 2.47, *p* = 0.015) (Yuen's *t* test for trimmed means, Table S3). Unlike leaf and storage traits, growth (AGR) did not differ between high‐ and low‐vitality trees (*p* = 0.181). Also, the mean annual height increment was similar for both vitality classes and varied between 5.2 and 6.5 cm for high‐vitality trees and between 4.4 and 5.7 cm for low‐vitality individuals (Figure S4).

### 3.3 Influence of LAR, AGR, and NSC pool on species' juvenile survival time

Effects of tree age, shoot growth (leaf + branch mass per year), species identity, LMF, and the interaction between tree age and shoot growth were significant for the response variables LAR, AGR, and NSC in the MANCOVA model, based on a Pillai test (Figure S4). For every 1% increase in tree age, LAR decreased by 1.33% and AGR by 0.74%, while NSC increased by 9.37% (Table 3). For every 1% increase in shoot growth rate, LAR significantly decreased by 0.04% while AGR and NSC were enhanced by 0.83% and 0.82%, respectively. Compared with *A. platanoides*, *A. pseudoplatanus* had a lower leaf area ratio and growth rate but a higher storage demand (although not to a significant degree). Compared with *A. platanoides*, *F. sylvatica* was associated with a higher LAR (not significant), significantly slower growth and lower NSC storage if all other predictors were held constant. A 1% change in LMF significantly affected all response variables, positively influencing LAR by 0.83% and negatively affecting AGR and NSC, by 0.89% and 0.49%, respectively. The model featured a low standard deviation of the residuals (prediction errors or RMSE) except for NSC, and a high goodness‐of‐fit (*R* ^2^ = 0.83–0.98).

**TABLE 3:** MANCOVA summary: effect of explanatory variables tree age, shoot growth rate, species identity, leaf mass fraction (LMF), and interaction between tree age and shoot growth rate on leaf area ratio (LAR), absolute growth rate (AGR), and content of non−structural carbohydrates (NSC)

Response	Predictors	*p*‐Value	*T*‐stat	Coef	CI	*SE*	RMSE	*R* ^2^
Ln(LAR)	(Intercept)	**<.001**	125.66	3.92	3.87–3.99	0.03	*0.26*	.*88*
	Tree age	**<.001**	−3.84	−0.01	(−0.02)–(−0.01)	0.00		
	Ln(shoot growth rate)	.**002**	−2.99	−0.04	(−0.07)–(−0.01)	0.01		
	*A. platanoides*	reference						
	*A. pseudoplatanus*	.**024**	−2.27	−0.10	(−0.18)–(−0.01)	0.04		
	*F. sylvatica*	.385	0.87	0.04	(−0.05)–(−0.13)	0.04		
	Ln(LMF)	**<.001**	32.86	0.83	0.78–0.88	0.02		
	Tree age × ln(shoot growth rate)	.**001**	3.33	0.00	0.00–0.01	0.00		
					*F*(6, 241) = 268.6, *p*‐value: <2.2e−16			
Ln(AGR)	(Intercept)	.849	−0.19	−0.00	3.87–3.99	0.02	*0.41*	.*98*
	Tree age	.**006**	−2.75	−0.01	(−0.02)–(−0.00)	0.02		
	Ln(shoot growth rate)	**<.001**	76.24	0.83	(−0.07)–(−0.01)	0.01		
	*A. platanoides*	reference						
	*A. pseudoplatanus*	.126	−1.53	−0.05	(−0.18)–(−0.01)	0.03		
	*F. sylvatica*	**<.001**	−5.48	−0.19	(−0.05)–(−0.13)	0.03		
	Ln(LMF)	**<.001**	−45.49	−0.89	0.78–0.88	0.01		
	Tree age × ln(shoot growth rate)	**<.001**	−5.78	−0.00	0.00–0.00	0.00		
					*F*(6, 241) = 2270, *p*‐value: <2.2e−16			
Ln(NSC)	(Intercept)	**<.001**	−27.09	−2.65	3.87–3.99	0.09	*0.85*	.*83*
	Tree age	**<.001**	8.15	0.09	(−0.02)–(−0.01)	0.01		
	Ln(shoot growth rate)	**<.001**	18.41	0.81	(‐0.07) – (‐0.01)	0.04		
	*A. platanoides*	reference						
	*A. pseudoplatanus*	.146	1.46	0.19	(−0.18)–(−0.01)	0.13		
	*F. sylvatica*	**<.001**	−3.41	−0.48	(−0.05)–(−0.13)	0.14		
	Ln(LMF)	**<.001**	−6.14	−0.49	0.78–0.88	0.08		
	Tree age × ln(shoot growth rate)	**<.001**	−6.62	−0.03	0.00–0.01	0.00		
					*F*(6, 241) = 224.4, *p*‐value: <2.2e−16			

Note

Bold marks significance of effect. Abbreviations: CI—0.95 confidence intervals, SE—standard error, RMSE—root‐mean‐square error or prediction error, R2—the variance of the response variable explained by the explanatory variables.

**FIGURE 2:**
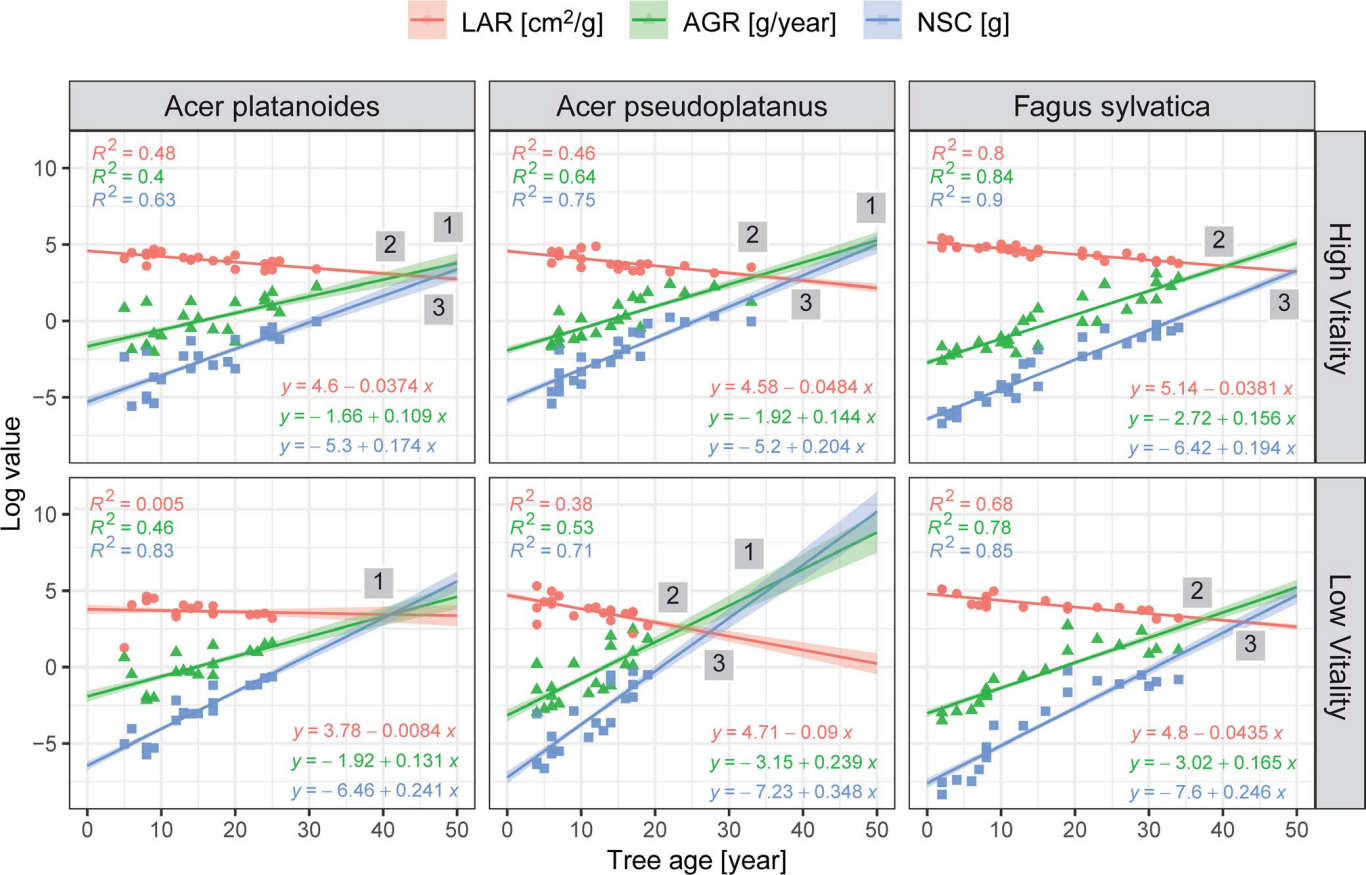
Linear regression predicting the development of LAR, AGR, and NSC with tree age: Dots represent observed values, bands represent 0.95 confidence intervals (CI), point 1 indicates carbon limitation as a result of a trade‐off between AGR (growth) and NSC pool (storage), point 2 indicates a limiting LAR capacity to support growth, and point 3 indicates a limiting LAR capacity to support storage

The predictions of the MANCOVA model for LAR, AGR, and NSC over tree age suggest that juvenile trees of the three species are facing similar trade‐offs between investment to leaves (LAR), growth (AGR), and storage (NSC) (Figure 2). The decline in LAR over time is due to an increasing tree biomass, while the increase in AGR means that tree biomass is increasing at ever higher rates, in particular given the log scale of the vertical axis (Figure 2). In line with the increasing AGR, the absolute value of the NSC pool is also increasing, due to the growing parenchyma tissue. The relevant pattern in Figure 2 is, therefore, the difference in slope between LAR on the one hand and AGR and NSC on the other hand. Unfortunately, we lack data for trees in the height class >5 m (and thus of older age) and can only hypothesize that the trajectory of AGR and NSC would have continued to develop in a linear (on a log scale) manner or would have taken another trajectory. Another trajectory assumes a slowing of growth and/or storage, when both may reach a plateau. Still, the pattern emerging from Figure 2 is that, in high‐vitality trees of the same age, the two *Acer* spp. have a smaller LAR at their disposal (smaller LAR intercept values) than *F. sylvatica*, due to faster growth (higher AGR intercept value) than in *F. sylvatica*, and also need more storage than *F. sylvatica* (visible from the higher NSC intercept value in Figure 2).

This relationship is more evident in low‐vitality *Acer* spp. trees where LAR is lower or decreasing faster (steeper slope) than in high‐vitality regeneration while AGR and NSC increase faster. Low‐vitality *F. sylvatica* trees differ from high‐vitality trees in that they have a lower LAR and exhibit faster growth of AGR and NSC values, as indicated by the comparably steeper lines. The different rates of change in LAR, AGR, and NSC lead to a trade‐off point between AGR and NSC due to carbon limitation, which is reached earlier in low‐vitality trees than in high‐vitality trees (point 1, Figure 2). Carbon limitation occurs as a result of the ever‐shrinking capacity of LAR to support both growth (point 2) and storage (point 3), assuming no improvement of light availability during this development.

**FIGURE 3:**
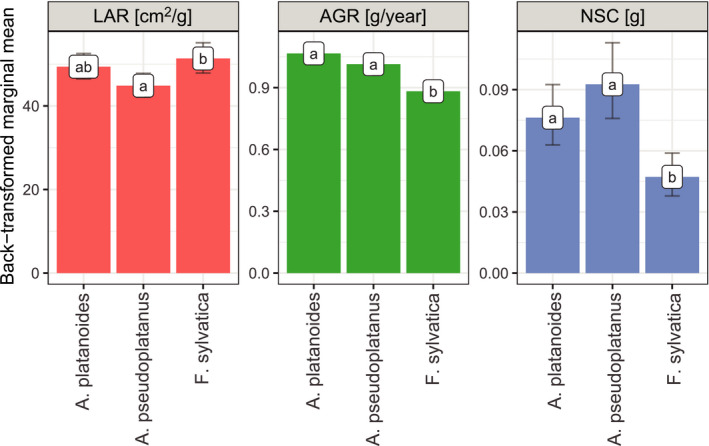
Pairwise comparison among species with the post hoc Tukey HSD test: Different letters indicate significant differences, whiskers represent 0.95 CI, and marginal mean assumes tree age = 14.3, shoot growth = 0.4 g/year, and LMF = 0.1

The three species have different allocation strategies, as confirmed by the predicted marginal mean of the MANCOVA model (assuming tree age = 14.1, shoot growth = 0.4 g, and LMF = 0.1). Compared with *A. platanoides* (Figure 3), *A. pseudoplatanus* had the lowest and *F. sylvatica* the highest marginal mean of LAR, although the difference was not significant. However, a significant difference was observed between the mean LAR of *A. pseudoplatanus* (44.85 cm^2^/g) and that of *F. sylvatica* (51.36 cm^2^/g, *p*‐value = 0.007). The high AGR of *A. platanoides* (1.07 g/year) did not differ from the moderate growth rate of *A. pseudoplatanus* (1.01 g/year), although the growth rate of both of these species was significantly higher than that of *F. sylvatica* (0.88 g/year, *p*‐value < 0.001). Compared with *A. platanoides* (0.08 g), mean NSC allocation was higher in *A. pseudoplatanus* (0.09 g) and lower in *F. sylvatica* (0.05 g, *p* < 0.001). In short, *A. platanoides* put growth at stake and maintained a moderate leaf area ratio and storage allocation, while *A. pseudoplatanus* invested in storage at the expense of allocation to leaves. At the same time, *F. sylvatica* sacrificed growth and storage to increase LAR.

## 4 DISCUSSION

### 4.1 Traits of shade tolerance and their impact on survival time

The aggregation of traits, rather than each trait alone, and their shift during ontogeny determine the performance and the persistence strategy of species occurring in the shade (Ameztegui et al., 2017; Gibert et al., 2016). Although LAR, AGR, and NSC pool are traits associated with shade tolerance (Valladares & Niinemets, 2008), they have not previously been combined to infer their impact on regeneration survival time. The potential trade‐off between growth and storage traits in juvenile trees growing in deep shade has not been shown so far (Palacio et al., 2014). In our study, the trade‐off between growth (AGR) and storage (NSC) at decreasing capacity of LAR can lead to a reduction in survival time under carbon limitation (point 1, Figure 2). Thus, our model can partly explain the long survival time of *F. sylvatica* in old‐growth deciduous forests.

The “carbon gain” concept is based on the assumption that shade‐tolerant species have a higher LAR, and therefore improved light interception, than shade‐intolerant species and thus a larger fraction of biomass is allocated to foliage (Bazzaz, 1979; Givnish, 1988). Indeed, in our study the marginal mean LAR of *F. sylvatica* was higher and declined more slowly during ontogeny compared with its competitors (Figure 2), confirming findings by other authors (Annighöfer et al., 2017; Niinemets, 1998). Such phenotypic plasticity can be explained by two factors: (a) low annual growth allows *F. sylvatica* to balance leaf area per unit mass and (b) the energy (glucose) required for building a unit of leaf area is lower in juvenile *F. sylvatica* than in *A. pseudoplatanus* (Petriţan et al., 2010). A failure to balance leaf area with growth leads to consistently higher mortality in seedlings of species with a higher relative growth rate than in seedlings with a lower relative growth rate in deep shade (Walters & Reich, 1996). Hence, *F. sylvatica* maintains a higher LAR for more efficient capture of diffuse light at a relatively “cheap” leaf construction cost.

According to several studies (Popma & Bongers, 1988; Walters & Reich, 1996), the growth rate of young trees in low light should be higher for shade‐tolerant species and lower for shade‐intolerant species. Our findings imply the opposite, however, as *F. sylvatica* grew slowly at a young age and focused biomass allocation to branches and leaves (horizontal growth), supporting the findings of Petriţan et al. (2010) and Collet et al. (2011). The negative relationship between growth and survival time in *F. sylvatica* for the first 70 years of its life was observed in studies by Di Filippo et al. (2012), Di Filippo et al. (2015). Similar relationships have been observed between the growth rate of conifers and lifespan in the first 50 years of life; that is, fast early growth was associated with decreased lifespan (Bigler, 2016).

Following the “defence and storage” concept, shade‐tolerant species partition a major fraction of photosynthates to internal stores at the expense of rapid growth (Kitajima, 1994; Kobe, 1997). In our study, *F. sylvatica* trees had a lower NSC content during slow growth compared with the less shade‐tolerant *Acer* spp., thus contradicting the “defence and storage” concept. Similar results as in our study were found for *Acer saccharum* (Kobe, 1997) and for evergreen shade‐tolerant species (Lusk & Piper, 2007; Piper et al., 2009); however, no difference has been reported for other deciduous species—*Castanea crenata* and *Quercus mongolica* (Imaji & Seiwa, 2010). On the one hand, early spring leaf‐out of *A. pseudoplatanus* compared with juvenile *F. sylvatica* produces more photosynthates before canopy closure (Vitasse, 2013). A high concentration of NSCs in *Acer* spp. may also reflect high levels of browsing and defoliation, as starch and sugars are used to survive periods of a negative net carbon balance after defoliation (Myers & Kitajima, 2007) or stem loss (Latt et al., 2000). On the other hand, faster‐growing juvenile trees increase total storage (Canham et al., 1999; Niinemets, 1998). In our study, both *Acer* spp. had higher AGR values than *F. sylvatica* and also maintained higher NSC content, while *F. sylvatica* showed the opposite (Figure 2).

Assuming that light had not improved and LAR reached its carrying capacity in carbon supply to growth (point 2) and storage (point 3, Figure 2), a tree can have two strategies: (a) slow down growth to save storage while keeping LAR high; or (b) deplete storage to maintain growth while decreasing LAR. Many empirical studies have shown that storage is prioritized over growth under carbon limitation (Weber et al., 2018; Wiley et al., 2013). A replenishment of a certain level of NSCs before growth leads to allocation of carbon first to storage and then to growth (Imaji & Seiwa, 2010; Weber et al., 2018). However, larger saplings require more storage to support the increasing operational costs of tissue maintenance and defense (Wiley & Helliker, 2012). This is why allocation to storage may increase disproportionately compared with allocation to growth (point 1, Figure 2), leading to a trade‐off between growth and storage.

Under environmental conditions leading to carbon limitation (long‐term deep shade or sustained severe defoliation), a trade‐off between storage and growth is possible (Palacio et al., 2014), causing carbon starvation and tree death (to the right of point 1, Figure 2) (Weber et al., 2018). Presently, it is unclear whether saplings deplete their NSC reserves before growth reduction or death in the shade. In an experiment by Weber et al. (2019), mortality of shaded *F. sylvatica* seedlings (0–60 cm tall) occurred after NSC concentrations in the stem dropped to approx. 3% (dry mass basis) after insect herbivore attack. In our study, the mean NSC concentration in the stem of low‐vitality *F. sylvatica* seedlings was 7.8% of dry mass for the same height class (comparison is valid if the method is the same). The mortality of *A. pseudoplatanus* happened at less than 1% (dry mass basis) NSC concentration in the study by Weber et al. (2018), while our low‐vitality *A. pseudoplatanus* seedlings maintained mean NSC levels of 9.7% (dry mass basis) for the same height classes. In our study, we did not observe growth reduction, as LAR was still able to support both growth and storage, hence lethal carbon starvation could not be assumed. However, unlike trees in the canopy, young trees do not have the opportunity to replenish NSC reserves in autumn (Hoch et al., 2003) because leaf senescence of trees and regeneration occurs at same time (Varsamis et al., 2019; Vitasse et al., 2009). Therefore, the NSC reserves of young trees may decrease for decades, eventually leading to tree death. We therefore conclude that the earlier crossing point of the AGR and NSC regression lines for the two *Acer* spp. indicates that carbon starvation occurs at a younger age compared with *F. sylvatica*.

With a slow AGR, species can have a longer survival time because the trade‐off between growth and storage is postponed in time. In our study, slower growth—indicated by low AGR values and low storage demand (indicated by low NSC values)—postponed this trade‐off, thus extending the survival time of *F. sylvatica* in both vitality classes compared with that of the two *Acer* spp., under the assumption of a linear development of AGR and NSC on a log scale. *Fagus sylvatica* of both vitality classes most likely do not experience the trade‐off (point 1, Figure 2) before the age of 50, while both *Acer* spp. in the two vitality classes are likely to approach this point at an age of 40–45 years. Taking into account that the average period of the first release to nearby canopy gaps is at 65 years for beech in Uholka (Trotsiuk et al., 2012), we suggest that *F. sylvatica* is able to balance LAR, NSC, and AGR trajectories and extend its survival time by at least two decades compared with the two *Acer* spp. Thus, this model can explain the overall dominance of *F. sylvatica* juveniles, the drop in the share of *Acer* spp. trees from 15% to 3% in DBH class 3–3.9 cm, and the absence of *A. platanoides* regeneration starting from 2 cm DBH in Uholka (Figure S5). *Acer* spp. of high vitality would have reached up to 5 cm diameter at root collar (corresponding to 3 cm DBH, based on a regression between DRC and DBH) and died if there was no change in light availability following a canopy opening, while beech would have grown up to 7 cm diameter at root collar (up to 5 cm DBH) and would have had a higher chance of survival until the first release into the canopy.

### 4.2 Species biomass allocation and tree vitality

Biomass allocation of trees with low and high vitality differed significantly in our study, reflecting different performance in shade. Biomass allocation patterns to leaves (LMF, leaf area, LAR) and storage pool (NSC) define vitality, whereas AGR and height increment do not and do not differ between low‐ and high‐vitality regeneration (Table S3). In addition, similar height increments for all species do not indicate shade avoidance (Henry & Aarssen, 2001). In previous studies, *F. sylvatica* saplings in the height class 201–500 cm featured greater leaf areas compared with *Acer* spp., while greater biomass allocation to branches in *F. sylvatica* led to larger crowns compared with the more slender form of *Acer* spp. (Annighöfer et al., 2017; Petriţan et al., 2009). Both *Acer* spp. studied here, even in the larger height classes, tend to grow long petioles over branches (Beaudet & Messier, 1998). Such kind of leaf display may be an adaptation to browsing (Modrý et al., 2004), enabling minimization of biomass loss by allocating less biomass to branches and leaves and concentrating it on the top to avoid self‐shading. In our study, *F. sylvatica* and *Acer* spp. of low vitality had a smaller leaf area and crown volume than high‐vitality trees. As a result of crown decline and reduced leaf area, the level of NSCs was reduced in trees with low vitality (Table S3), in agreement with previous research (Hartmann & Trumbore, 2016; Schönbeck et al., 2018).

### 4.3 Limitations

Although our model offers a plausible explanation for the regeneration growth processes, it is, nevertheless, linear and can thus be applied only to the period of intensive growth when response and explanatory variables are developing mostly linearly. Furthermore, it assumes equal annual AGR and shoot growth, which may be theoretically possible but in reality varies with tree age and size (Gibert et al., 2016). The model does not take into account NSC pool of branches and the seasonal variation, an aspect that might be needed for a better understanding of the annual storage balance. Moreover, NSC concentrations were scaled to the whole stem and root. This is justified for relatively small saplings with wood consisting almost entirely of sapwood but seems problematic for saplings with larger vertical and horizontal NSC gradients within the stem.

## 5 CONCLUSION

The proposed integrated model of shade tolerance explains the longer survival time of *F. sylvatica* juveniles compared with *Acer* spp. species in deep shade. It combines traits from the “carbon gain” (LAR and AGR) and the “defence and storage” (NSC) hypotheses. Despite mechanistic approaches for the explanation of trait development, the model leads to inferences about the survival time of young trees without its direct measurement. Due to shifts in the above three traits with increasing tree height and age, juvenile trees may increasingly face a trade‐off because a diminishing LAR becomes insufficient to produce the photosynthates needed to support both growth and storage. In this case, a tree can either reduce growth to retain storage, or deplete storage to achieve growth. The ability of a species to balance LAR, AGR, and NSC to postpone or avoid this trade‐off defines its shade tolerance and thus its regeneration survival time. *Fagus sylvatica* is able to minimize both AGR and NSC, maintaining a high LAR, while the two *Acer* spp. cannot reduce storage and/or growth. The increased storage in *Acer* spp. may be explained by early leaf‐out in spring and a focus on defense, sacrificing investment into leaf and branch biomass.

Our findings only partly confirm the “carbon gain” concept: beech optimizes carbon gain with an extensive leaf display, large crown volume, and slowly decreasing LAR, but it grows slowly in the shade. However, with only storage in focus, our results also cannot fully support the “defence and storage” concept, as shade‐tolerant *F. sylvatica* stores less NSCs than its competitors.

## CONFLICT OF INTEREST

None declared.

## AUTHOR CONTRIBUTIONS

**Roksolana Petrovska:** Conceptualization (equal); Data curation (lead); Visualization (lead); Writing‐original draft (lead). **Peter Brang:** Conceptualization (equal); Methodology (equal); Project administration (lead); Supervision (lead); Writing‐review & editing (equal). **Arthur Gessler:** Conceptualization (equal); Methodology (equal); Writing‐review & editing (equal). **Harald Bugmann:** Conceptualization (equal); Supervision (lead); Writing‐review & editing (lead). **Martina Lena Hobi:** Conceptualization (equal); Project administration (lead); Supervision (lead); Writing‐review & editing (equal).

## Supporting information

Supplementary Material

## Data Availability

Regeneration biomass (AGB & BGB), age, NSCs, and light: *Fagus sylvatica* and *Acer* spp.: https://doi.org/10.5061/dryad.05qfttf30.
