# Supplementary material for: Grow slowly, persist, dominate—Explaining beech dominance in a primeval forest
Source: Ecol Evol. 2021 Jul 7;11(15):10077–89. doi: 10.1002/ece3.7800 (PMC8328449; doi:10.1002/ece3.7800)
Supplement: Supplementary file 1 — Supplementary Material [file ECE3-11-10077-s001.pdf]

Grow slowly, persist, dominate – explaining beech dominance in a primeval forest

Petrovska R.[1,2], Brang P.[2], Gessler A.[2], Bugmann H.[2], Hobi M.[2]

ELECTRONIC SUPPLEMENTARY MATERIAL (ESM)

Table S 1. Mean and standard deviation (in brackets) of variables for high- and low-vitality trees (H: tree height class; Sp: species; N: number of trees; a_pla: Acer platanoides; a_pse: A. pseudoplatanus; f_syl: Fagus sylvatica; LMF: leaf mass fraction; LAR: leaf area ratio; AGR: absolute growth rate; NSC: pool of non-structural carbohydrates; ISF: indirect site factor).

High vitality

				Leaf traits				Growth		Storage	ISF
H	Sp	N	Age	Leaf area [cm^2]	Crown volume [m^3]	LMF	LAR [cm^2/g]	AGR [g/yr]	Shoot growth rate [g/yr]	NSC [g]	%
0–10 cm	a_pla	7	6.43 (1.90)	113.8 (98.55)	0 (0)	0.17 (0.08)	107.83 (54.53)	0.18 (0.17)	0.05 (0.08)	0.01 (0.01)	3.25 (0.01)
	a_pse	7	7 (1.10)	113.57 (80.78)	0 (0)	0.14 (0.08)	113.23 (86.97)	0.22 (0.09)	0.02 (0.01)	0.01 (0.01)	3.37 (0.01)
	f_syl	5	3.2 (1.1)	46.64 (26.14)	0 (0)	0.17 (0.10)	118.36 (50.76)	0.14 (0.07)	0.02 (0.01)	0 (0)	3.34 (0.02)
11–20 cm	a_pla	5	9 (2.58)	214.96 (157.41)	0 (0)	0.14 (0.08)	91.34 (86.11)	0.24 (0.11)	0.03 (0.02)	0.06 (0.08)	2.13 (0.01)
	a_pse	5	9.6 (2.61)	195.9 (181.29)	0 (0)	0.16 (0.09)	102.79 (62.71)	0.22 (0.10)	0.05 (0.05)	0.01 (0.01)	3.13 (0.01)
	f_syl	6	5.17 (5.23)	131.08 (100.64)	0 (0)	0.25 (0.08)	185.68 (71.41)	0.17 (0.05)	0.04 (0.01)	0 (0)	2.29 (0.01)
21–35 cm	a_pla	6	11 (4.86)	702.9 (708.17)	0.01 (0.01)	0.1 (0.05)	77.55 (44.67)	0.94 (0.97)	0.1 (0.10)	0.06 (0.05)	2.82 (0.01)
	a_pse	8	11.43 (3.95)	597.27 (478.04)	0.08 (0.21)	0.14 (0.08)	89.76 (59.90)	1.49 (2.69)	0.14 (0.15)	0.12 (0.17)	3.05 (0.01)
	f_syl	7	8.57 (3.15)	434.4 (209.95)	0.01 (0.01)	0.22 (0.04)	154.46 (43.02)	0.4 (0.28)	0.1 (0.09)	0.02 (0.02)	3.9 (0.01)
36–60 cm	a_pla	6	12 (4.77)	565.78 (205.09)	0.02 (0.02)	0.07 (0.03)	49.69 (7.37)	1.12 (0.69)	0.11 (0.10)	0.07 (0.04)	3.27 (0.01)
	a_pse	5	14 (5.70)	583.24 (200.23)	0 (0.01)	0.08 (0.04)	54.61 (34.40)	0.95 (0.36)	0.17 (0.19)	0.09 (0.07)	2.35 (0)
	f_syl	8	15.25 (5.70)	890.84 (523.19)	0.06 (0.05)	0.14 (0.06)	91.87 (53.49)	0.85 (0.65)	0.18 (0.13)	0.08 (0.07)	2.76 (0.01)
61–90 cm	a_pla	6	12 (3.85)	869.92 (316.08)	0.04 (0.03)	0.08 (0.02)	49.64 (15.77)	1.8 (1.18)	0.15 (0.07)	0.14 (0.05)	3.34 (0.01)
	a_pse	6	13.17 (5.04)	1005.35 (646.07)	0.02 (0.02)	0.1 (0.03)	63.85 (24.95)	1.46 (0.88)	0.28 (0.23)	0.20 (0.15)	3.01 (0.01)
	f_syl	7	15.57 (2.82)	2137.21 (1050.28)	0.22 (0.17)	0.13 (0.04)	87.04 (25.23)	1.63 (0.78)	0.44 (0.26)	0.15 (0.06)	2.32 (0.01)
91–130 cm	a_pla	7	18.57 (6.35)	2187.04 (1362.52)	0.13 (0.07)	0.06 (0.03)	41.45 (19.11)	3.03 (1.07)	0.47 (0.40)	0.30 (0.2)	3.68 (0.01)
	a_pse	7	22.29 (6.60)	1672.26 (773.25)	0.2 (0.27)	0.05 (0.01)	33.2 (11.80)	2.41 (0.87)	0.24 (0.13)	0.25 (0.18)	3.02 (0.01)
	f_syl	5	23.2 (3.77)	5521.82 (2445.22)	1.15 (0.62)	0.11 (0.04)	77.41 (28.50)	3.33 (1.84)	0.81 (0.51)	0.31 (0.18)	3.35 (0.02)
131–200 cm	a_pla	5	18.5 (7.72)	3310.88 (1655.16)	0.46 (0.14)	0.08 (0.02)	58.79 (35.55)	3.37 (1.21)	0.45 (0.23)	0.31 (0.11)	2.42 (0.01)
	a_pse	4	19.75 (3.20)	2936.3 (933.39)	0.38 (0.34)	0.06 (0.03)	35.9 (6.73)	4.18 (1.20)	0.58 (0.37)	0.64 (0.42)	2.4 (0.01)
	f_syl	8	26.57 (7.55)	9400.06 (4678.56)	2.08 (1.01)	0.08 (0.03)	49.54 (12.78)	8.21 (4.11)	2.32 (1.57)	0.81 (0.44)	2.47 (0)
	a_pla	8	23.62 (6.44)	7418.56 (5260.26)	2.02 (2.48)	0.06 (0.03)	34.69 (10.24)	9.1 (4.99)	0.99 (0.57)	1.14 (1.35)	2.33 (0.01)

Low vitality

				Leaf traits				Growth		Storage	ISF
H	Sp	N	Age	Leaf area [cm^2]	Crown volume [m^3]	LMF	LAR [cm^2/g]	AGR [g/yr]	Shoot growth rate [g/yr]	NSC [g]	%
0–10 cm	a_pla	6	6.5 (1.87)	57.82 (25.08)	0 (0)	0.08 (0.05)	59.89 (46.8)	0.54 (0.83)	0.01 (0.01)	0.01 (0.01)	2.91 (0.01)
	a_pse	7	5.33 (1.21)	30.87 (11.98)	0 (0)	0.13 (0.1)	92.22 (66.16)	0.09 (0.07)	0.03 (0.05)	0 (0)	2.82 (0.01)
	f_syl	6	4.5 (2.81)	29.85 (15.78)	0 (0)	0.09 (0.06)	134.07 (125.99)	0.09 (0.06)	0.01 (0)	0 (0.01)	3.48 (0.02)
11–20 cm	a_pla	4	7.5 (1.91)	110.72 (47.24)	0 (0)	0.11 (0.07)	76.42 (49.96)	0.41 (0.56)	0.02 (0.01)	0.02 (0.03)	2.68 (0.01)
	a_pse	7	8 (3.46)	75.51 (66.98)	0 (0)	0.08 (0.07)	52.92 (40.82)	0.21 (0.16)	0.01 (0)	0.01 (0.01)	2.78 (0.01)
	f_syl	6	5.33 (2.34)	51.67 (33.19)	0 (0)	0.08 (0.03)	91 (48.97)	0.13 (0.08)	0.02 (0.01)	0 (0)	2.4 (0.01)
21–35 cm	a_pla	6	11.17 (2.99)	260.5 (151.91)	0 (0.01)	0.07 (0.03)	53.22 (23.27)	0.49 (0.22)	0.03 (0.02)	0.05 (0.05)	3.04 (0.01)
	a_pse	5	10 (3.39)	104.98 (39.49)	0 (0)	0.08 (0.05)	52.34 (33.10)	0.26 (0.12)	0.02 (0.01)	0.02 (0.02)	2.23 (0)
	f_syl	9	10.78 (3.63)	143.59 (52.52)	0.01 (0.01)	0.09 (0.06)	65.91 (47.46)	0.33 (0.31)	0.03 (0.05)	0.02 (0.02)	3.09 (0.02)
36–60 cm	a_pla	6	14.83 (5.23)	459.37 (289.89)	0.02 (0.03)	0.04 (0.03)	32.56 (15.82)	1 (0.35)	0.08 (0.07)	0.05 (0.04)	2.85 (0.01)
	a_pse	6	9.5 (4.55)	233.15 (211.24)	0 (0)	0.06 (0.05)	48.58 (48.84)	1 (0.95)	0.04 (0.04)	0.04 (0.03)	2.03 (0.01)
	f_syl	7	15 (3.7)	301.49 (213.16)	0.02 (0.02)	0.09 (0.09)	53.09 (49.84)	0.56 (0.22)	0.06 (0.05)	0.03 (0.02)	2.72 (0.01)
61–90 cm	a_pla	6	14 (3.52)	431.25 (134.92)	0.03 (0.02)	0.04 (0.02)	28.03 (10.26)	1.3 (0.73)	0.09 (0.05)	0.04 (0.04)	2.21 (0.01)
	a_pse	7	14 (4.62)	606.84 (267.41)	0.01 (0.01)	0.06 (0.04)	29.46 (12.27)	1.63 (0.56)	0.12 (0.10)	0.18 (0.13)	2.59 (0.02)
	f_syl	4	23.25 (6.6)	2571.28 (1677.05)	0.17 (0.12)	0.11 (0.04)	71.2 (28.60)	1.62 (0.82)	0.45 (0.36)	0.2 (0.04)	2.27 (0)
91–130 cm	a_pla	6	12.83 (5.04)	672.9 (402.56)	0.05 (0.06)	0.04 (0.03)	29.79 (25.22)	2.89 (1.87)	0.34 (0.38)	0.1 (0.11)	2.42 (0.01)
	a_pse	5	15 (5.66)	1349.3 (1527.17)	0.04 (0.07)	0.05 (0.03)	31.85 (16.22)	2.81 (1.63)	0.26 (0.21)	0.29 (0.18)	2.25 (0.01)
	f_syl	5	22.2 (3.42)	2260.35 (1416.53)	0.39 (0.36)	0.05 (0.02)	47.14 (26.40)	2.62 (1.11)	0.54 (0.29)	0.2 (0.17)	1.78 (0.01)
131–200 cm	a_pla	5	16.4 (5.08)	1900.4 (532.16)	0.11 (0.05)	0.12 (0.16)	35.23 (14.64)	3.7 (1.27)	0.43 (0.46)	0.25 (0.13)	3.31 (0.01)
	a_pse	5	16.8 (2.28)	1592.15 (510.09)	0.22 (0.2)	0.05 (0.02)	32.64 (18.91)	3.5 (1.20)	0.24 (0.12)	0.37 (0.16)	2.23 (0)
	f_syl	7	25 (3.27)	4576.51 (2707.01)	2.05 (1.51)	0.06 (0.03)	32.92 (19.86)	6.28 (3.04)	1.27 (0.65)	0.45 (0.41)	2.12 (0.01)
	a_pla	6	22.17 (5.49)	3080.32 (1585.94)	0.42 (0.43)	0.04 (0.02)	29.21 (15.18)	5.64 (1.96)	0.34 (0.24)	0.46 (0.42)	3.24 (0.01)

4

201–500 cm	a_pse	5	22.8 (3.83)	5919.78 (1323.89)	1.25 (0.49)	0.06 (0.02)	35.29 (10.39)	7.84 (2.43)	1.1 (0.14)	0.93 (0.51)	2.1 (0.01)	5	20.6 (7.77)	3736.18 (2882.62)	0.43 (0.46)	0.03 (0.02)	23.05 (17.59)	10.01 (5.91)	0.97 (0.98)	1.75 (1.49)	2.53 (0.02)
	f_syl	5	30.6 (3.91)	25982.97 (6692.77)	9.25 (8.07)	0.08 (0.01)	42.38 (10.02)	19.97 (7.89)	6.19 (2.87)	1.92 (1.23)	2.28 (0.01)	5	29 (5.87)	6804.82 (4783.76)	3.15 (4.3)	0.05 (0.02)	23.61 (11.06)	10.88 (7.54)	1.77 (1.73)	1.25 (1.4)	2.81 (0.01)

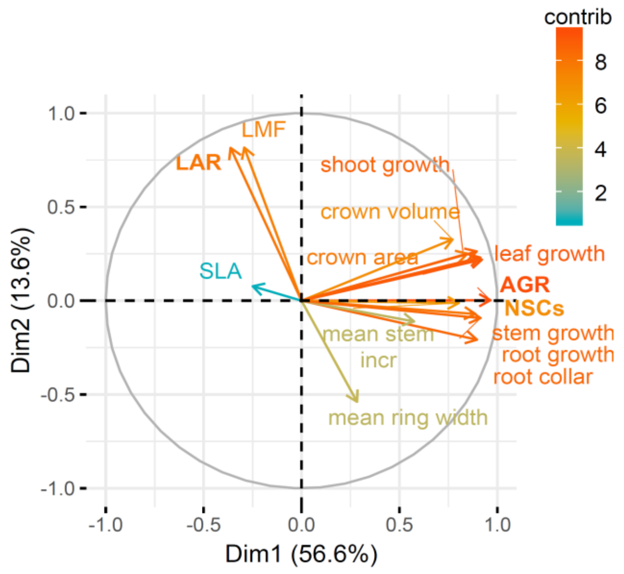

Figure S 1. Leaf, growth and storage traits' contributions to principal components. Variables in blue contribute the least and those in red the most to the two principal components.

Table S 2. Variables' contributions to the two principal components.

Trait	Variable	PC1, %	PC2, %
Growth	AGR [g/year]	10.96	0.00
Leaf	Leaf area [cm^2]	10.00	2.35
Growth	Root growth rate [g/year]	9.85	0.42
Growth	Leaf growth rate [g/year]	9.83	2.60
Growth	Diameter at root collar [cm]	9.46	2.13
Growth	Shoot growth rate [g/year]	9.43	3.39
Growth	Stem growth rate [g/year]	9.30	0.25
Leaf	Crown area [m^2]	8.46	3.09
Storage	NSC [g]	7.59	0.01
Leaf	Crown volume [m^3]	7.01	5.24
Growth	Mean H incr. [cm/y]	3.89	0.60
Leaf	LAR [cm^2/g]	1.55	32.6
Leaf	LMF	1.01	32.7
Growth	Mean ring [µm]	0.95	14.2
Leaf	SLA [cm^2/g]	0.73	0.29

Table S 3. Results of Yuen's t-test for trimmed means between the trees of low and high vitality.

Traits	Var.	Yuen's t-test	p-value
**Leaf**	LAR	t (205.71) = 4.92	<0.001
	LMF	t (219.37) = 5.88	<0.001
**Storage**	NSC	t (178.60) = 2.47	0.015
**Growth**	AGR	t (221.21) = 1.34	0.181

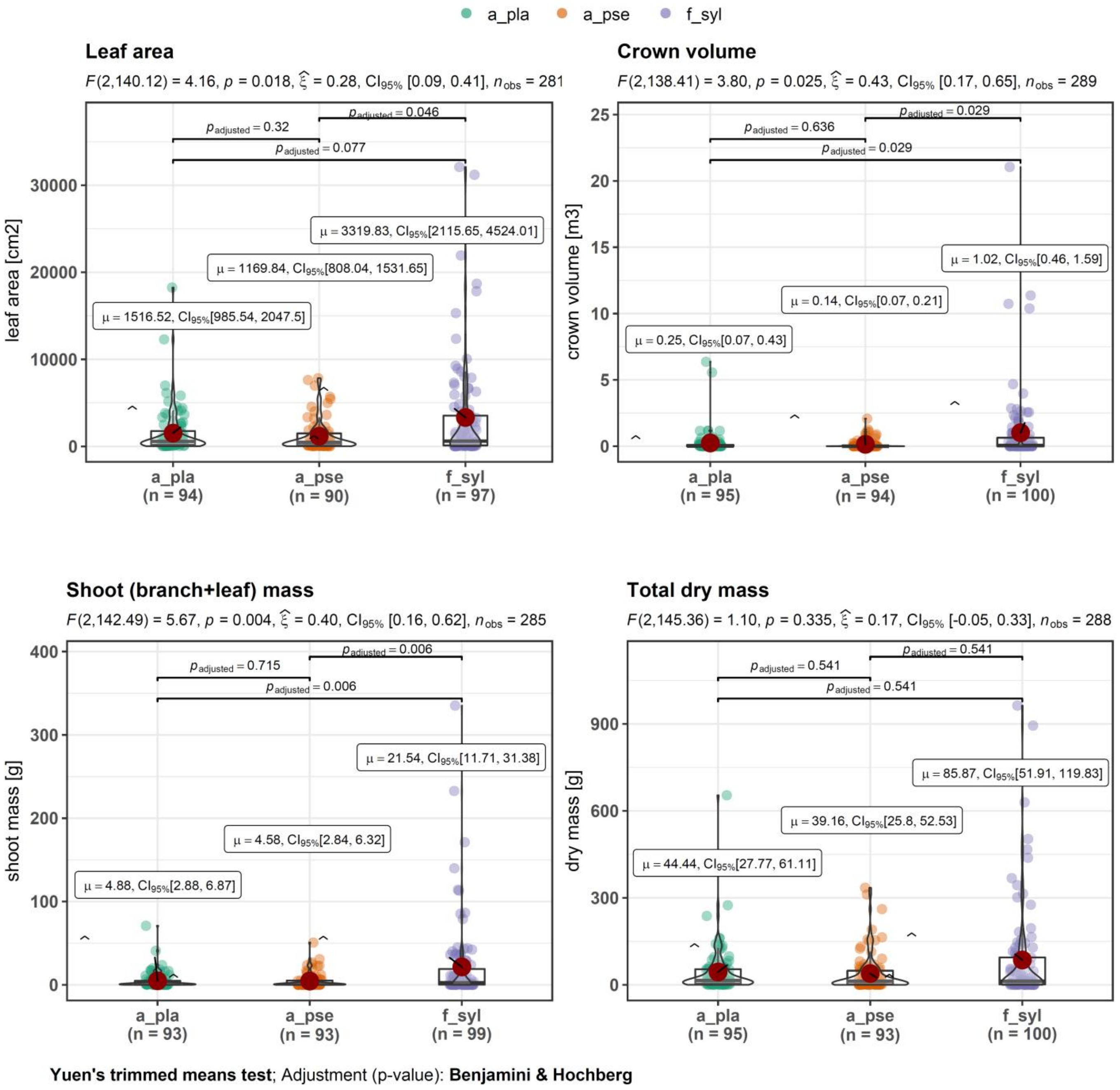

Figure S 2. Results of the heteroscedastic two-way ANOVA for all trees: leaf area, crown volume, shoots and total dry mass for Acer platanoides (a_pla), A. pseudoplatanus (a_pse) and Fagus sylvatica (f_syl). Red dots represent mean values.

S1.1 MANOVA AND 50 × 50 MANOVA

Result of the Pillai Test are presented in Table S4 (upper part). The significance of explanatory variables was

additionally checked with a 50 × 50 MANOVA with rotation testing (R package *ffmanova*, Langsrud and Mevik 2019), a variant of classical MANOVA modified to handle highly correlated responses using a Monte Carlo simulation (10,000 iterations) enabling exact significance testing under multivariate normality (Table S4, lower part). The unbalanced design was handled by using a variant of Type II sums of squares (Langsrud 2003), which is insensitive to the ordering of the model terms and to scale changes.

Table S4. Results of the classical MANCOVA showing the Pillai test for ANOVA type III and 50 × 50 MANOVA with bootstrapping with ANOVA type II. Vitality and height classes are pooled.

**MANCOVA, Pillai test**	**Df**	**Pillai**	**Approx F**	**num Df**	**den Df**	**p-value**
Tree age	1	0.96766	2383.77	3	239	< 2.2e-16
Ln(shoot growth)	1	0.94576	1389.09	3	239	< 2.2e-16
Species	2	0.73665	46.65	6	480	< 2.2e-16
Ln(LMF)	1	0.93178	1088.12	3	239	< 2.2e-16
Tree age × ln(shoot growth)	1	0.20909	21.06	3	239	3.848e-12

**50 × 50, Hotelling-Lawley test**	**Df**	**exVarSS**	**nPC**	**nBU**	**exVarPC**	**exVarBU**	**p-value**
Tree age	1	0.00897	3	0	1.00	1	7.56e-
Ln(shoot growth)	1	0.25897	3	1	0.94	1	< 2e-16***
Species	2	0.01321	3	0	1.00	1	3.12e-
Ln(LMF)	1	0.25086	2	1	0.95	1	< 2e-16***
Tree age × ln(shoot growth)	1	0.01554	3	0	1.00	1	1.04e-

exVarSS: explained variances calculated from sums of squares summed over all responses, nPC: number of principal components used for testing, nBU: number of principal components used as buffer components, exVarPC: variance explained by nPC components, exVarBU: variance explained by (nPC+nBU) components.

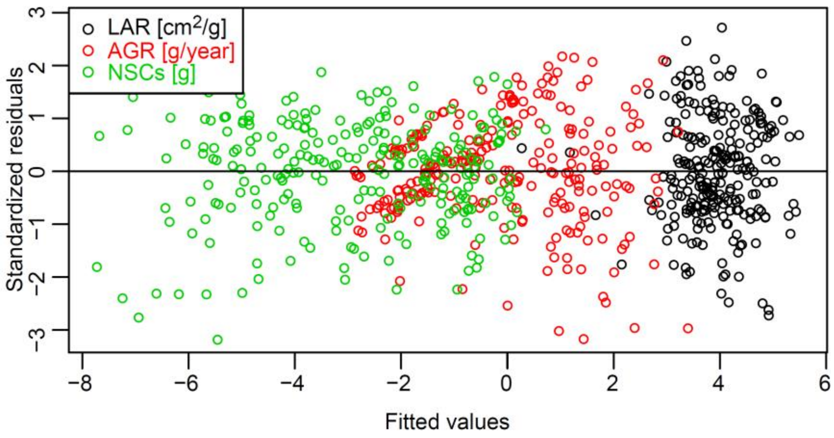

Figure S 3. Standardised residuals of the MANCOVA model for three response variables: LAR (leaf area ratio), AGR (absolute growth rate) and NSC (non-structural carbohydrates).

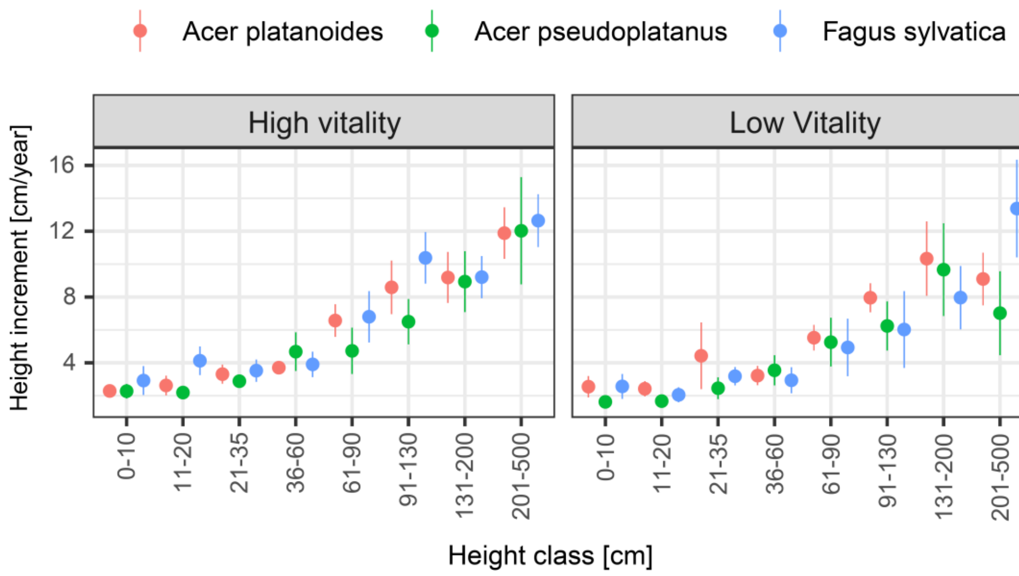

Figure S 4. Mean (dot) and standard error (whiskers) of tree height increment according to species and height class for trees of high and low vitality.

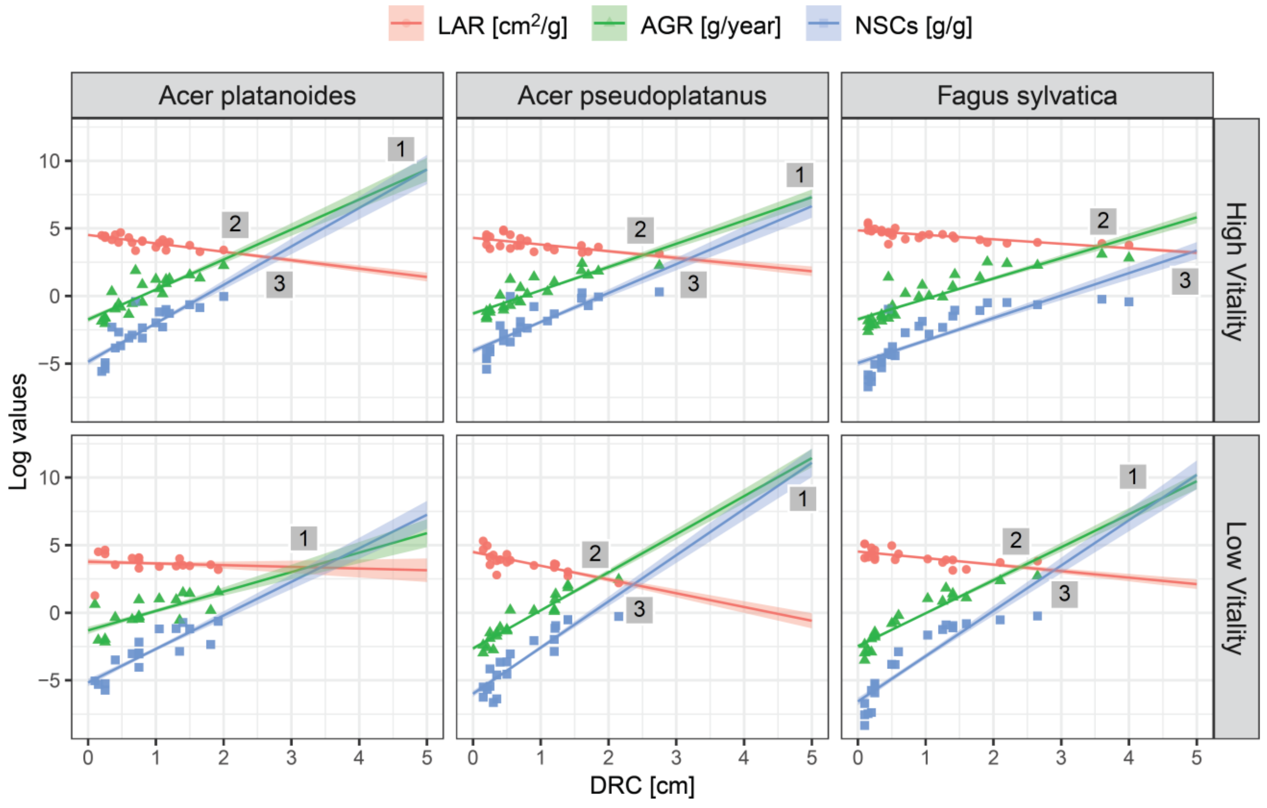

Figure S 5. LAR (leaf area area), AGR (absolute growth rate) and NSC (non-structural carbohydrates) development with diameter at root collar (DRC). Dots: fitted values, shaded bands: 0.95 confidence intervals.
